# Phylogeography and Prevalence of Hemoparasites (Apicomplexa: Eucoccidiorida) in Galápagos Marine Iguanas, *Amblyrhynchus cristatus* (Reptilia: Iguanidae)

**DOI:** 10.3390/ani12091142

**Published:** 2022-04-28

**Authors:** Jessica Scheibel, Joan Garcia-Porta, Galo Quezada, Alejandro Ibáñez

**Affiliations:** 1 Zoological Institute, Technische Universität Braunschweig, 38106 Braunschweig, Germany; 2 Department of Biology, Washington University in St. Louis, St. Louis, MO 63130, USA; j.garcia-porta@wustl.edu; 3 Dirección Parque Nacional Galápagos, Puerto Ayora 200102, Ecuador; galofernandoquezada@gmail.com; 4 Department of Ecology and Vertebrate Zoology, Faculty of Biology and Environmental Protection, University of Łódź, 90-237 Łódź, Poland

**Keywords:** hemoparasites, genetic diversity, prevalence, biogeographic patterns, ticks, infection status assessment, marine iguanas

## Abstract

**Simple Summary:**

It is estimated that a vast diversity of parasites populate our planet, many of them still to be discovered. Blood parasites (hemoparasites) of the phylum Apicomplexa may infect both vertebrates and invertebrates. Here, we used molecular methods to characterize the diversity of hemoparasites in the emblematic Galápagos marine iguana. We examined major island populations of marine iguanas across the Galápagos archipelago, performing the largest biogeographical study on the topic in this species. In addition, ticks were collected from marine iguana’s body to screen for hemoparasites. Our genetic analysis revealed two distinct clusters of hemoparasites belonging to *Hepatozoon* and/or *Hemolivia*. The occurrence of hemoparasites differed dramatically across islands, indicating that some populations may have mechanisms to reduce infection rates. The infection status of ticks did not match with their marine iguana hosts, suggesting that this method cannot be used to reliably know whether or not marine iguanas are infected. Given that apicomplexan blood parasites may cause important zoonotic diseases, studies like this one focusing on the relationship between parasites, their hosts and transmitting vectors, may help to shed light on the underlying mechanisms that wild animals employ to cope with pathogen infection.

**Abstract:**

Parasitism is among the most common forms of coexistence of organisms of different species. Hemoparasites live in the bloodstream of the host where they complete different life-cycle stages. Members of the phylum Apicomplexa constitute a large portion of all hemoparasites infecting reptiles and their parasite transmitting vectors, including arthropods. In this study, we carried out a survey and molecular identification of hemoparasites in blood samples of the iconic Galápagos marine iguana (*Amblyrhynchus cristatus*). Major island populations of marine iguanas were sampled to examine large-scale biogeographic patterns of parasite diversity and prevalence. Nested PCRs were used to amplify segments of the 18S rRNA-gene of hemoparasites. Furthermore, ticks attached to marine iguanas were collected and analyzed in the same way to assess their potential use as a non-invasive method for the detection of hemoparasites in vertebrate host species. PCR products were sequenced and a phylogenetic analysis was carried out showing the presence of two genetically distinct clusters of hemoparasites, one more commonly distributed than the other one, belonging to the genera *Hepatozoon* and/or *Hemolivia* (Apicomplexa: Eucoccidiorida). Overall, 25% of marine iguanas were infected by hemoparasites. However, infection rates varied strongly among particular island populations (from 3.45% to 50%). Although marine iguanas are an extremely mobile species that has colonized all islands in the Galápagos archipelago, parasite occurrence was not related to geographical distance, suggesting that dispersal behavior has a minor role in parasite transmission. On most islands, females tended to have higher infection rates than males, but this relationship was only significant on one island. Overall, ticks and marine iguanas had similar prevalence and diversity of parasites. However, the infection profiles of ticks and their corresponding hosts (marine iguanas) did not mirror one another, indicating that this method cannot be used reliably to assess marine iguana infection status. Interestingly, we found that hemoparasite prevalence in marine iguanas and ticks tended to be positively correlated across islands. Our results indicate that certain populations of marine iguanas may have special mechanisms and adaptations to cope with parasite infection. In addition, other factors such as vector density, anthropogenic-related activities or the immunological state of marine iguanas could potentially affect the striking variation in hemoparasite prevalence across island populations.

## 1. Introduction

Apicomplexa is a phylum of obligate eukaryotic endoparasites with complex life cycles [1]. A distinct characteristic of apicomplexan parasites is the alternation of sexual and asexual reproduction in particular generations [2]. Both vertebrates and invertebrates are used as hosts [3]. Among vertebrates, reptiles are hosts to a large number of species of apicomplexan parasites. Despite its great prevalence in reptiles, the actual extent of their diversity is actually unknown [4]. This is especially problematic in threatened or vulnerable host species, as, if they go extinct, all parasites associated to them may also go extinct, reducing the diversity of parasites and ultimately hampering the correct functioning of ecosystems [5]. 

Here, we studied the hidden diversity of apicomplexan parasites in a highly vulnerable species: the marine iguanas of the Galápagos (*Amblyrhynchus cristatus* Bell, 1825). This species, endemic from the Galápagos Islands, is threatened by human pressure, introduced predators and environmental fluctuations, such as “El Niño” events [6,7,8].

Even though many aspects of marine iguana’s morphology, physiology and genetics have received much attention [9,10,11,12,13,14,15,16,17], studies on blood parasites have been largely neglected. Previous morphological assessments revealed the presence of haemogregarines in marine iguanas from the island of Santa Cruz, potentially akin to *Hepatozoon* Miller, 1908 [18]. However, to our knowledge, there is no comprehensive study examining the diversity, distribution and prevalence of these parasites across the Galápagos Islands. 

In this study, we fill this gap, by characterizing the diversity and phylogeography of apicomplexan hemoparasites in marine iguanas and exploring large-scale biogeographical patterns of hemoparasitic infection. For that purpose, we sampled marine iguanas from eleven islands of the Galápagos archipelago, including a total of 13 distinct sites—covering most of the described subspecies [19]. We focused on the phylum Apicomplexa since these parasites are widespread in reptiles and have been previously detected in marine iguanas [4,18]. The prevalence of hemoparasites may vary highly among reptilian host species [20,21]. Such variation may occur in species living in distinct habitats but also in isolated populations. For instance, in lizards of the genus *Podarcis* Wagler, 1830, the occurrence of hemogregarines is much greater in island populations than in continental ones [22]. However, a remarkable degree of variation in hemoparasite infection rates may occur even between populations of the same species living in geographical proximity or in similar environments (e.g., *Hepatozoon* in Spanish terrapins [23]). Marine iguanas present an amphibious lifestyle, presenting specialized foraging and locomotor adaptations to the marine environment, and are thought to have a great dispersal behaviour [24,25,26,27]. Such mobility has allowed marine iguanas the colonization of all islands in the Galápagos archipelago, a fact that contrasts with land iguanas (genus *Conolophus* Fitzinger, 1843)—the sister lineage of *Amblyrhynchus*—that have a much more restricted distribution than marine iguanas. Thus, we expected that such high dispersal ability could affect the transmission of parasites across islands. Migrants from neighbouring islands have been found in some populations, indicating that these are not totally isolated units [12]. In any case, migration of individuals is more likely to happen between closely located islands than between far and remote ones. In this scenario, we would expect a more similar prevalence and diversity of parasites in marine iguana populations from nearby sites than those from distant locations. 

Another aim of the study was to examine the possible relation between hemoparasite vectors, such as ticks, and their vertebrate hosts (marine iguanas). For that purpose, we also surveyed the ticks collected from the marine iguanas’ bodies to screen for the presence of parasites. Since hemoparasites are tick-transmitted, we expected a high prevalence of hemoparasites in ticks originating from marine iguana populations with high infection rates. In addition, we examined the potential use of ectoparasites (ticks) as a non-invasive method to detect hemoparasites in their vertebrate hosts (marine iguanas). Previous research had shown that there might be distinct groups of ticks infecting marine iguanas [28,29]. The most obvious and common ticks belong to genus *Amblyomma* Koch, 1844, these ticks are usually attached to soft ventral parts of the body, like the cloaca and legs [28,29]. Another group of less prominent ticks infecting *A. cristatus* is *Ornithodoros* Koch, 1844—usually infesting marine iguanas during the night or when these are in crevices [29]. Ticks are hematophagous ectoparasites and potential vectors transmitting haemogregarines in reptiles [30,31]. We predicted that hemoparasite dynamics should be closely linked in ticks and marine iguanas, and thus, a similar diversity and prevalence of parasites is expected in both organisms. In addition, we compared blood and tick samples obtained from the same host iguanas to test whether or not these match their infection status, and to explore the potential use of ticks as a reliable method to assess marine iguana infection condition.

The main aims of the study were: (1) examine differences in hemoparasite diversity and prevalence in major island populations of *A. cristatus*; (2) test how geographical distance and parasite occurrence are related in a very mobile reptilian species; (3) preliminary exploration of the possible link between ticks and marine iguanas in terms of hemoparasite prevalence; (3) test a potential method to evaluate the presence or absence of hemoparasites in marine iguanas hosts. 

## 2. Materials and Methods

### 2.1. Sampling 

**Figure 1:**
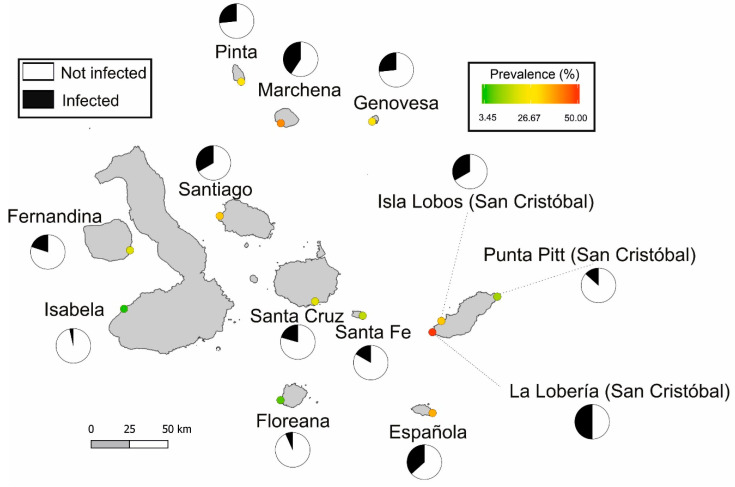
Map of the Galápagos Islands showing the different sampling sites of this study. Hemoparasite infection prevalence for marine iguanas (blood samples) in each site is shown. In the pie charts, black and white represent, respectively, the proportion of infected and non-infected marine iguanas (*n* = 390). Sampling sites are marked with circles. Colors filling the circles reflect prevalence (%) according to the scale.

Blood samples were obtained from a total of 390 marine iguanas (*A. cristatus*) from eleven islands of the Galápagos archipelago during December 2015 and January 2016. Marine iguanas were sampled typically at one site per each island with the exception of San Cristóbal, where samples were collected at three sites (“La Lobería”, “Isla Lobos” and “Punta Pitt”). The two northernmost islands (Darwin and Wolf) were not considered in this study, due to logistical reasons. In Figure 1, a map with all sampling sites is shown. The map was generated in QGIS version 3.6.2 (QGIS Geographic Information System. https://www.qgis.org, accessed on 30 January 2022), using a base layer shapefile freely available at http://www.statsilk.com/maps/download-free-shapefile-maps (accessed on 29 January 2022). Whenever it was possible, iguanas were sexed on the basis of external features (e.g., dorsal crest scales/spines and femoral pores [19]). From the total of marine iguanas sampled, 197 were classified as males, 159 as females, 30 as subadults (sex could not be determined). In 4 individuals, the sex was not recorded. 

Blood samples were taken from the tail vein at a point between the cloaca and the tail tip. Samples were stored in buffer (100 mM Tris, 100 mM EDTA, 2% SDS) until DNA extraction. In addition, ticks were collected from a total of 75 marine iguanas (at least one tick per individual). All the ticks collected from a single marine iguana individual (i.e., “a tick sample”) were pooled together in the same tube for further analysis. According to the external aspect, the ticks sampled in this study were hard ticks (Ixodidae). Although ticks collected in this study were likely *Amblyomma*, the most prominent ticks in marine iguanas [29,32], no microscopic identification of the ticks was carried out, and thus, this must be taken as a tentative identification. 

In this study, we did not assess the abundance of ticks—i.e., ticks were not counted; thus, we did not account for that factor. Both blood and tick samples were subsequently processed in the same way, to screen for parasites.

All our sampling was performed during the day. Samples were collected under the authorization of the Galápagos National Park and Ministerio del Ambiente de Ecuador (PC-08-15 and MAE-DNB-CM-2016-0043), according to the legal regulations of the country.

### 2.2. Molecular Detection of Hemoparasites

Genomic DNA extraction in marine iguana blood samples and in the ticks was carried out similarly as in [33]. After several steps in the laboratory [33], the DNA was resuspended in 50 µL dH_2_O. Nested PCRs were performed with the genomic DNA to detect hemoparasite DNA. For the first PCR, the pair of primers HEMO1 and HEMO2 was used to separate hemoparasite DNA from the host’s DNA (*A. cristatus*) [34]. HEMO1 is a specific primer for haemogregarines and HEMO2 is a specific primer for Apicomplexa. The first PCR reaction was composed of: 2.50 µL 5× buffer, 0.25 µL 10 mM dNTPs, 0.30 µL HEMO1, 0.30 µL HEMO2, 8.05 µL dH_2_O, 0.10 µL GoTaq-Polymerase, 1 µL DNA-extract. The PCR was carried out with an initial denaturation of 94 °C for 180 s, followed by 40 cycles of 94 °C for 30 s, 48 °C for 30 s and 72 °C for 60 s, and a final extension of 72 °C for 10 min. The nested PCR consisted of the same components as the first PCR, but a different pair of primers, HepF300 and HepR900 [21], was used to amplify a partial sequence of the 18S rRNA of the parasite gene. The reaction was carried out with 1 µL of the product of the first PCR. The PCR was carried out with an initial denaturation of 94 °C for 180 s, followed by 45 cycles of 94 °C for 30 s, 61 °C for 30 s and 72 °C for 60 s, and a final extension of 72 °C for 10 min. The success of the nested PCR was proven with gel electrophoresis (1% agarose). The products of the nested PCR were purified and Sanger-sequenced. 

The software CodonCode Aligner (CodonCode Corporation) and MEGA5 [35] were used for quality control and to assemble and edit contigs. To identify hemoparasite species, all sequences were BLASTed (BLASTN [36]) against the NCBI database (nucleotide collection, https://www.ncbi.nlm.nih.gov/nucleotide/, accessed on 22 February 2022), restricting our search to the Phylum Apicomplexa, retrieving a maximum of 50 hits per sequence. The BLAST search relied on an expected threshold of 0.05, a word size of 28, and a match and mismatch score of 1 and −2, respectively. Of all BLAST hits, we downloaded all sequences belonging to different taxa, which provided a phylogenetic context external to the study system. We also downloaded from NCBI sequences of the distantly related genera: *Lankesterella* Labbé, 1899, *Caryospora* Léger, 1904, *Eimeria* Schneider, 1875 and *Choleoeimeria* Paperna and Landsberg, 1989, which served as outgroups [37,38,39,40].

All these sequences were aligned with the sequences produced by this study by means of the software MAFFT [41] (available in https://www.ebi.ac.uk/Tools/msa/mafft/, accessed 22 February 2022) using default settings. 

Poorly aligned regions were removed by means of GBlocks [42] using low stringency options (number of positions of the alignment was 352). After removing from the alignment all duplicate sequences, we built a phylogenetic tree in a maximum likelihood framework by means of IQ-TREE2 [43,44], determining the best-fitting model by means of ModelFinder [45] and 1000 replicates of ultrafast bootstrap to assess supports. All other IQ-TREE search parameters were left by default.

### 2.3. Statistical Analysis

We calculated the overall prevalence of blood parasites as the percentage of infected marine iguanas in the total number of individuals tested. The prevalence for each population was calculated. A Pearson’s Chi-square test was carried out to examine the association between island of origin and prevalence. Afterwards, a logistic regression was performed using *glm()* with family = “binomial” to examine which populations have statistically a higher probability of being infected by hemoparasites. The population with the lowest prevalence was taken as a reference level (i.e., Isabela was set up as a reference level, see Results). 

Based on sampling site geographic coordinates, a geographical distance matrix was calculated using R function *earth.dist* (package *fossil* [46]), as previously described [47]. Function *decostand*, R package *vegan* [48], was used to calculate proportional abundances for infected and non-infected marine iguanas per population. Then, function *dist* was used to compute a distance matrix based on these values. In order to test whether migration events tend to homogenize parasite abundance—i.e., if closer island populations have similar parasite prevalence—a mantel test was performed including geographic and parasite abundance distance matrices. 

In addition, we explored whether parasite occurrence was sex-specific by using Fisher exact tests separately for each population. These analyses were performed with a subset of 356 individuals for which sex was available. To explore a possible effect of sex-biased sampling on parasite occurrence, a Spearman correlation was performed between sex ratio (M:F) and parasite prevalence of each population.

Prevalence of hemoparasites was calculated in ticks as described above. A Fisher exact test was performed to test whether hemoparasite prevalence differed across islands. Then, we performed Spearman correlations to test whether parasite prevalence in marine iguanas and ticks were related. First, we used all islands in this analysis; however, we repeated this test excluding populations with extremely low sample size of ticks (see Results). In addition, to test whether tick infection mirrored marine iguana host infection, a McNecmar’s paired test was carried out. The analyses were run using the software Statistica (Statsoft) and R software [49] using the interface R studio. 

## 3. Results

### 3.1. Molecular Phylogenetics of Blood Parasite

**Figure 2:**
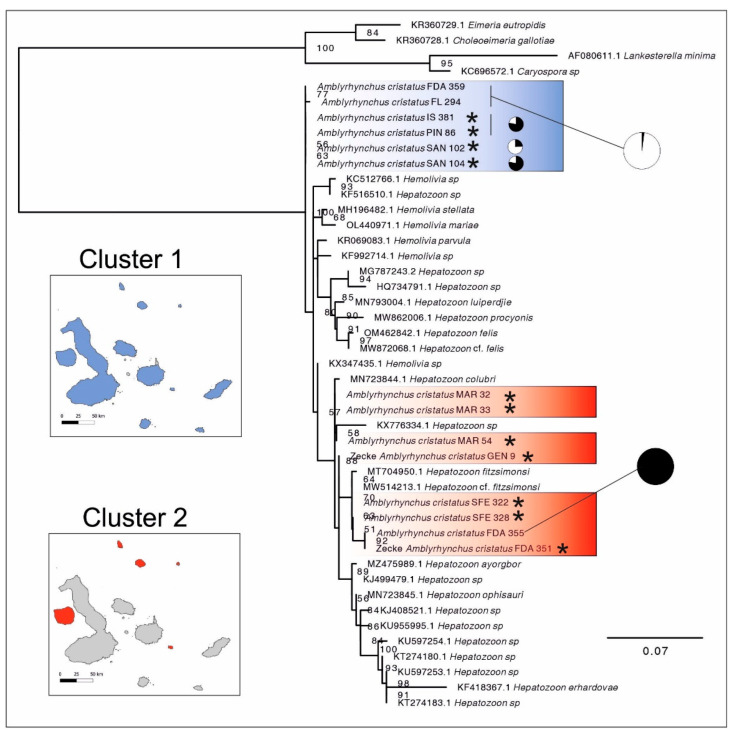
Phylogenetic tree including unique (selected) sequences generated in this study together with GenBank retrieved sequences. Samples from this study are highlighted with blue (cluster 1) and red (cluster 2). Distribution for the two clusters is shown. Pie charts represent the proportion of the first 50 BLAST hits for *Hepatozoon* (black) and *Hemolivia* (white) for selected sequences (i.e., short sequences in cluster 2 are not shown). Short sequences (<242 bp) are marked with an asterisk *. Genbank numbers and genus/species name are shown only for retrieved sequences. For samples from this study, GenBank accession numbers and other metadata are shown in Supplementary File S2. Bootstrap values (higher than 50) are shown. The scale bare represents substitutions per site.

A total of 120 samples (98 blood samples of marine iguanas and 22 ticks) contained partial sequences of hemoparasites (sequence length range; minimum–max: 215–354 bp). Our phylogenetic tree reveals the existence of two main parasite clades in the Galápagos Islands. One was widespread in all islands (cluster 1), and the other restricted in Genovesa, Marchena, Pinta, Fernandina and Santa Fe (cluster 2). Although precise identification was not possible, our molecular phylogeny suggests that cluster 1 could be likely *Hemolivia* Petit et al., 1990, while cluster 2 is nested within *Hepatozoon* (Figure 2). Results from the BLAST against GenBank sequences showed congruent results with that (see Supplementary File S1). Short sequences from cluster 1 included BLAST hits matching both *Hepatozoon* and *Hemolivia* (see Supplementary File S1 and Figure 2). However, this could be an artefact of the short size of these sequences.

### 3.2. Marine Iguana Blood Parasite Prevalence and Geographical Variation

Out of the 390 blood-sampled marine iguanas, 98 (overall prevalence = 25%) were infected with *Hepatozoon* or *Hemolivia*. There was no single population free of blood parasites. The lowest rate of infection was observed in the population of Isabela, located in the west of the Galápagos archipelago (3.45%). Iguanas from La Lobería (San Cristóbal) had the highest prevalence (50%) (see Table 1 and Figure 1 for an overview of the population prevalence). The proportion of infected and non-infected marine iguanas differed between islands and populations (Pearson’s Chi-square test: *χ*^2^ = 34.54, df = 12, *p*-value < 0.001). In addition, the results of the logistic regression revealed that marine iguana individuals from seven populations had statistically a higher probability of being infected with respect to the island of Isabela, that was set as a reference level since it had the lowest prevalence (see Table 2). Therefore, the probability of individual infection was statistically higher than the reference level in marine iguanas from Genovesa (*p* = 0.035), Pinta (*p* = 0.035), Santiago (*p* = 0.015), Isla Lobos—San Cristóbal (*p* = 0.015), Española (*p* = 0.01), Marchena (*p* = 0.007) and La Lobería—San Cristóbal (*p* = 0.002). The rest of the island did not significantly differ from the baseline (all *p* > 0.07; see Table 2). Concerning the distribution of the two groups of hemoparasites, we found that cluster 1 (majority of sequences, a total of 86 marine iguana blood samples), was widespread in all islands (at least one marine iguana infected in each population). In contrast, cluster 2 (included 12 marine iguana samples) was restricted to the northern islands (Genovesa, Marchena and Pinta) as well as Fernandina and Santa Fe (see Table 1 and Figure 2). A mantel test between a distance matrix based on the proportional abundances of infected and non-infected iguanas against a geographical distance matrix showed no relationship between both matrices (Mantel statistic *r* = 0.022; *p* = 0.43). Thus, differences in the proportion of infected and non-infected individuals among sites were not driven by geographic distance. 

Furthermore, a subset of the dataset (*n* = 356) was used to examine potential sexual patterns on parasitic infection across islands. We found that, in most of the island populations, females had higher infection rates than males did (see Table 3). Indeed, except Isla Lobos—San Cristóbal and Santa Fe, all the other populations had proportionally more females infected than males. However, this difference was significant only in one island (Fernandina; Fisher’s exact test *p* = 0.017). In addition, our sampling was biased toward males. Except Pinta and Genovesa that had more females than males sampled and Fernandina that had equal numbers of both sexes, all the other populations were male-skewed. However, sex ration was not correlated to parasite prevalence (*p* = 0.76). 

### 3.3. Tick Hemoparasites and Comparison to the Infection Status of Marine Iguanas

**Figure 3:**
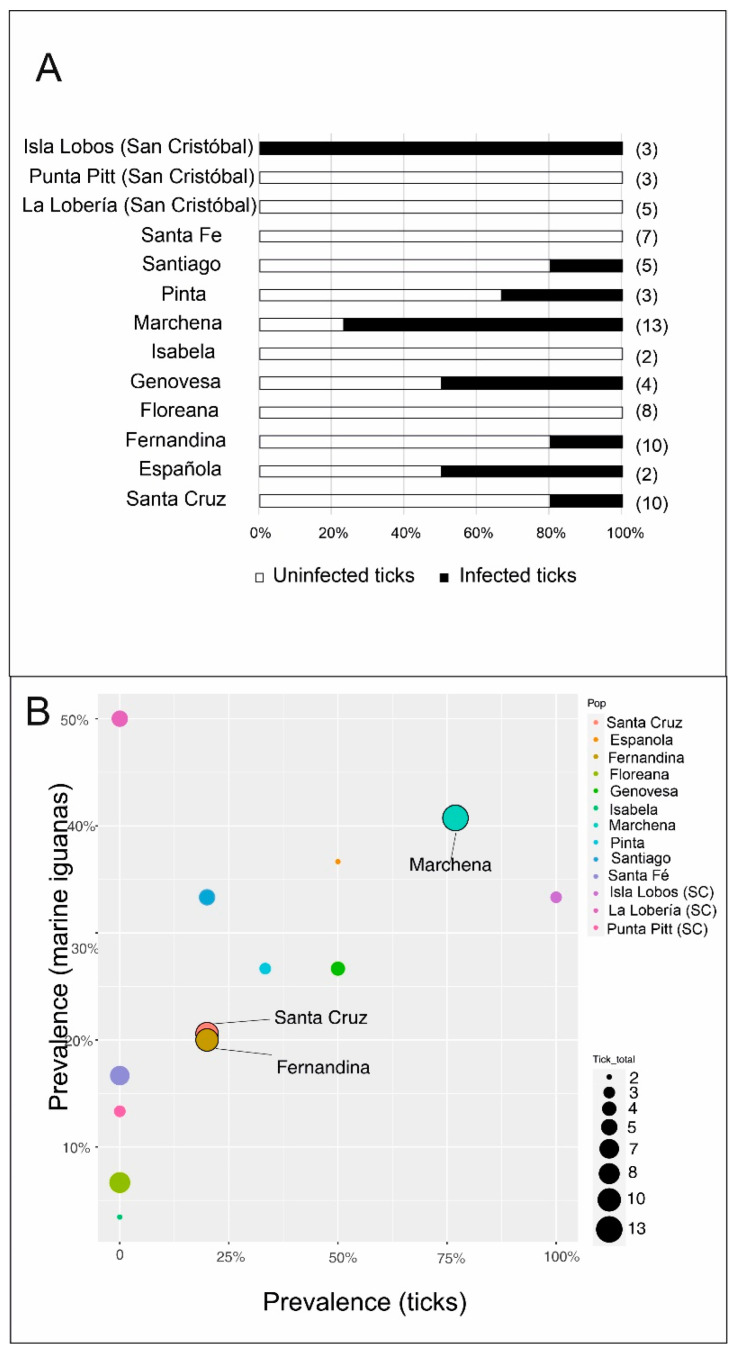
Prevalence of hemoparasites in tick samples (i.e., one or more ticks were collected for each “tick sample”, see Materials and Methods for clarification). (**A**) Percentage of infected and uninfected tick samples per each site. In brackets, the total number of tick samples collected. (**B**) Scatterplot showing prevalence values for marine iguanas (*y*-axis) and prevalence values for tick samples (*x*-axis). The size of the point is according to the sample size of tick samples. Most reliable estimates of hemoparasite prevalence in ticks—i.e., populations with ten or more samples—are marked with the name.

A total of 22 out of the 75 tick samples had 18S rRNA gene sequences from *Hepatozoon* or *Hemolivia* (overall prevalence = 29%). The number of tick samples (i.e., number of marine iguanas for which one or more ticks were sampled) collected per island ranged from 2 to 13 (Figure 3A shows the percentage of infected and uninfected tick samples per island). Tick prevalence was associated to site of origin (Fisher’s exact test, *p* < 0.001). Prevalence in tick samples varied greatly among sites and included five sites for which hemoparasites were not detected at all (see Figure 3A). In contrast, all ticks collected in Isla Lobos (San Cristóbal) had parasites. However, this comparison needs to be taken carefully due to the small number of tick samples collected per site (typically less than ten, and in some cases, even lower than three). The largest number of samples was collected in Santa Cruz, Fernandina and Marchena, for which the observed prevalence was ~20% in the two first islands and close to 77% in the third one (see Figure 3A). Both hemoparasite prevalence in marine iguanas and ticks was positively correlated across islands (Spearman rank correlation, rho = 0.60, *p* = 0.03; see Figure 3B for a visualization of the data). However, after excluding populations with extremely low tick sample size (i.e., less than three tick samples: Española and Isabela), the relationship was not significant, but only a positive trend was observed (rho = 0.52, *p* = 0.10; see Figure 3B). As with marine iguana samples, the two clusters of hemoparasites were present in ticks, and the vast majority of positive infected samples belonged to cluster 1 (see Supplementary File S2). 

From the 22 infected ticks, 63.6% were collected from marine iguanas which were uninfected with hemoparasites. Only 36.4% (8/22) of the infected ticks were collected from infected marine iguanas. As a general trend, we found that infection status of the ticks was not associated with that of their marine iguana hosts (McNemar’s Chi-squared test, *χ*^2^ = 0.375, df = 1, *p* = 0.5403). An overview of the paired tick-iguana data can be found in Table 4.

## 4. Discussion

### 4.1. Phylogeography of Apicomplexan Parasites in the Galápagos Islands 

In this study, we looked at DNA partial sequences to determine the presence of Apicomplexan parasites in marine iguanas. Genetic analysis demonstrated that, at the least, two clusters or lineages are present in marine iguanas. According to our results, these sequences belong to the genera *Hemolivia* and/or *Hepatozoon*. The genetic identity of these hemoparasites is uncertain [50,51], and we therefore only tentatively identify these as representatives of *Hepatozoon* and *Hemolivia*. In any case, all the hemoparasites found in this study belong to the order Eucoccidiorida (Apicomplexa). In the Galápagos land iguanas (genus *Conolophus*), *Hepatozoon* has been identified morphologically [52]. In our study, we found that parasite genetic cluster 2 was grouped within a large group that includes other *Hepatozoon* species with well-supported clades (see Figure 2). However, sequences from cluster 1 had a high similarity with *Hepatozoon* sp. MIG2 (JQ080303.1) [53] (see Supplementary File S1). Partial sequence of the 18S rRNA gene from *Hepatozoon* sp. MIG2 was isolated out of the stomach contents of the blood-feeding mosquito *Aedes taeniorhynchus* (Wiedemann, 1821)—supposedly fed on marine iguana blood—although our assessment of this sequence through a BLAST search showed a high similarity with species of the genus *Hemolivia*. Although the primers applied in this study are typically used for the detection of *Hepatozoon*, they may also amplify for *Hemolivia* in reptiles and their ectoparasite ticks [54,55]. Our phylogenetic tree supports that finding and indicates the presence of two distinct clusters of hemoparasites in marine iguanas that could belong either to *Hepatozoon* and/or to *Hemolivia.*

### 4.2. Biogeographical Patterns of Hemoparasite Infection across Islands

About one out of four marine iguanas were infected with hemoparasites. However, the prevalence varied remarkably across marine iguana populations, with some islands presenting low parasite prevalence (i.e., inferior to 10%—Isabela and Floreana), while other sites harbouring populations with 40–50% of all individuals being infected (i.e., Marchena and La Lobería-San Cristóbal; see Figure 1). A large geographic and inter-island population variation of blood parasite prevalence is seen in other lizard species as well. For instance, lizards of the species *Teira dugesii* (Milne-Edwards, 1829), were screened for *Hepatozoon* in the Azores, showing infection rates of ~30% in the main island (Graciosa) to less than 5% in the relatively smaller islet of Praia [56]. The amphibious lifestyle and adaptation to the sea render marine iguanas as extremely mobile species able to travel long distances. For instance, in San Cristóbal, hybrids originating of the inter-mix from local and nearby islands’ populations (e.g., Santa Cruz and Española) have been found indicating that marine iguanas are able to travel between islands [12]. However, despite the striking variation in parasite prevalence among islands, our mantel test revealed no correlation between geographical distance and differences in parasite abundance among sites or populations. This indicates that individual colonization events do not drive parasite transmission, and instead parasite prevalence is mainly changing at a local scale. 

Further evidence for this conclusion comes from the sampling performed at the island of San Cristóbal, in which three different sites were screened in this study. The results showed that prevalence values varied within the same island, showing striking differences ranging from 50% (in La Lobería) to only 13.33% in Punta Pitt, while Isla Lobos with 33.33% was somewhere in between (see Table 1). Both La Lobería and Isla Lobos belong to the same subspecies (*A. c. mertensi*) and are closely located, but still present a notable variation in the proportion of infected individuals, adding support to the hypothesis that other factors, rather than geographic proximity, affect parasite abundance. Interestingly, La Lobería is the closest (2–3 km) of the three sites to the main urban area in the island (i.e., Puerto Baquerizo Moreno), followed by Islas Lobos (8–10 km) and the remote area of Punta Pitt, situated around 40–50 km from this point (see Figure 1). How human-related activities may affect parasite-host relationships are still not well understood [57,58]. Some studies have shown that blood parasite prevalence is lower in urban habitats than in the natural ones [59,60]. However, a meta-analysis found that species living in the cities are at higher risk of parasite transmission [61]. Here, our data for the island of San Cristóbal are in line with this argument. Taking Isabela as a baseline (population with the lowest prevalence), we observed that both Isla Lobos and La Lobería statistically differed from this reference, while this was not the case of Punta Pitt (see Table 2). Potentially, iguanas from La Lobería and Isla Lobos could be under higher stress levels related to human disturbance (e.g., touristic activities) carried out at the main city of this island that could affect the health state of the individuals [62]. In contrast, the difficult access to the remote area of Punta Pitt could make marine iguanas from this site less subjected to anthropogenic pressures. However, the Galápagos Islands are extremely protected by the Ecuadorian laws as well as by the Galápagos National Park, and most of the territory remains uninhabited. In fact, in our study, most of the sampled islands are uninhabited and only four have stable human settlements (Santa Cruz, San Cristóbal, Isabela and Floreana). If we look at the whole picture, there seems to be no clear pattern on hemoparasite prevalence between islands with and without human populations. For instance, both the Isabela and Floreana islands have human settlements but have very low hemoparasite prevalence (less than 10%). However, it is worth mentioning that our sampling site in Isabela island is roughly 50 km far from the main urban area. In addition, the island of Floreana has only rural areas instead of urban ones and an estimated human population below 200 [63]. Therefore, we could assume that the anthropogenic pressure in these two island sampling sites is relatively low and perhaps comparable to other islands free of human settlements. Most of the human population in the Galápagos is concentrated in Santa Cruz (main city: Puerto Ayora, ~12,000 inhabitants) and San Cristóbal (Puerto Baquerizo Moreno, around 6500 inhabitants) [63]. A rough comparison between La Lobería (San Cristóbal) and Santa Cruz shows that they have quite uneven prevalence (i.e., 50 and ~20%, respectively), and indeed, only La Lobería differed from the reference level. In addition, marine iguanas from Española and Marchena were among the highest prevalence (in both cases more than 35%), and these are remote islands without any human settlements, thus challenging the hypothesis that anthropogenic activities could favor the spreading of hemoparasites. However, our current sampling design is not the most appropriate to test this hypothesis, and this issue should be tested in future studies. 

In almost every population, females tend to have higher infection rates than males, but this was only significant in one island (i.e., Fernandina; see Table 3). This result could be related to the lek-mating system of marine iguanas, in which females congregate in rocky areas along the coastline in order to mate with territorial males [64,65]. One hypothesis is that the aggregation of females in highly dense clusters could facilitate hemoparasite transmission via tick infestation (see below, next subsection for a discussion). Our sampling was sex-biased (e.g., in general terms, more males were sampled than females). Even though we acknowledge that a balanced sampling would get better prevalence estimates, we found that sex ratio was independent of island parasite prevalence (*p* = 0.76) and suggest a relatively small impact on our study. Thereby, no clear pattern between the excess in the number of males respect to females (or vice versa) and the prevalence of the site was observed. For example, the two islands with the highest male-skew sampling, Isabela and Marchena, showed strikingly disparate prevalence (3.45% and 40.74%, respectively). However, we also note that an island-balanced sampling design would get more accurate estimates of parasite prevalence.

Overall, our study shows that parasitic infection rate varies both across islands as well as at a local scale, suggesting that one or more mechanisms might be mediating parasite-resistance at a geographical level. Despite that marine iguanas are a highly mobile species able to swim and dive in the sea and have colonized all major and minor islands of the Galápagos, geographical distance across islands has no effect on homogenizing infection rates. Other factors such as urbanization or anthropogenic pressures seem to not have an obvious impact on parasite prevalence, but future studies should test this hypothesis by using a different approach than our study—e.g., by performing intensive sampling in multiple points within an island to see if there is gradient on parasite occurrence, taking as a reference the main urban area of the island. 

### 4.3. Tick Hemoparasites and Marine Iguana Host Infection

Ticks had an overall prevalence of hemoparasites similar to that of marine iguanas (overall prevalence: 29% and 25% respectively). The total number of tick samples collected was much reduced compared to marine iguana’s samples, including several populations for which prevalence estimates should be taken carefully due to the low sample size (see Figure 3). Thus, although a positive tendency between prevalence in marine iguanas and ticks was observed across islands, this result needs to be interpreted carefully due to the abovementioned limitations. Despite that, in three populations (Marchena, Santa Cruz and Fernandina), the number of tick samples is large enough to get reliable estimates of hemoparasite abundance (see Figure 3). While in Fernandina and Santa Cruz tick infection prevalence were similar to marine iguana from the same sites (around 20%), in Marchena, 10 out of 13 tick samples (~77%) were infected—that is substantially higher than the observed prevalence for marine iguanas (~41%). One potential explanation could be that vector abundance differs geographically, being more common in some islands than others. Marine iguanas are territorial animals and several individuals tend to cluster in leks during the mating season [64,65]. Such reproductive system could facilitate easy dispersal of ticks, able to quickly colonize new hosts in these iguana-dense clusters, thus enhancing infestation rates [29]. In this scenario, it might be possible that populations with high tick densities would uphold high transmission rates of *Hepatozoon* and *Hemolivia*. While this hypothesis could explain why hosts and vectors present similar infection rates in Fernandina and Santa Cruz, the large disparity in Marchena remains an open question and could suggest a potential mechanism of marine iguanas from this island to avoid or resist against tick-borne parasitic infections. A limitation of our study is that tick abundance was not measured (e.g., ticks were not counted). Thus, whether some island populations have a higher density of ticks that could trigger elevated hemoparasite prevalence needs to be examined in further studies. 

The infection profiles from marine iguanas and from the ticks did not mirror one another at an individual level. In other words, a tick infected with *Hepatozoon* or *Hemolivia* did not necessarily suggest an infection of the host marine iguana with the same hemoparasite. Both *Hepatozoon* and *Hemolivia* use reptiles as intermediate hosts, while ticks are definitive hosts that can also act as vectors [55,66]. Although these parasites are tick-transmitted, our results indicate that the use of ticks to evaluate parasitic infection in marine iguana hosts is not suitable. This was especially evident when assessing whether positive tick samples match the infection status of the corresponding marine iguana host (only in ~36% of the ticks—iguana paired samples, taking into account all infected ticks). A limitation of this part of the study is that tick abundance was not assessed—ticks were not counted and this factor was not controlled in this study. If this potentially could have biased our comparison among individuals is unknown, but we must acknowledge that limitation. Thus, we conclude that, overall, this method cannot unambiguously assess marine iguana infection status. However, future studies should include ectoparasite abundance or density as a factor to corroborate our findings. 

## 5. Conclusions

Molecular evidence indicates the existence of apicomplexan blood parasites belonging to *Hepatozoon* and/or *Hemolivia*, in marine iguanas and their ticks. At least two distinct lineages or genetic clusters of hemoparasites are spread in marine iguanas across the Galápagos archipelago. A large biogeographic variation of parasite prevalence was observed among marine iguana island populations, but such disparity is independent of the geographical distance among sites. Female iguanas tended to be more likely to be infected than males in most of the islands, but a balanced sampling design is necessary to conclude whether infection rates are sex-specific across populations. Although overall prevalence in ticks and marine iguanas was similar, the use of ticks as a non-invasive method to assess individual host infection seems unsuitable in this species. 

These results indicate that some populations of marine iguanas may respond differently to parasitic infection by showing reduced infection rates, but the mechanism to cope with these parasites remains unknown. Further research examining patterns of vector density and distribution, as well as considering other factors such as individual health state or the impact of anthropogenic-related activities to understand parasite–host relationships in marine iguanas, is warranted. 

## Figures and Tables

**Table 1:** Prevalence of infection (%) and number of infected (both cluster 1 and cluster 2 are shown) and non-infected marine iguanas for each population (*n* = 390). Hemoparasite results refer to marine iguanas (i.e., blood samples). Island populations are sorted from lower to higher prevalence. Populations in which two distinct clusters have been found are highlighted in bold. SC = San Cristóbal.

Pop./Site	Subspecies	Longitude	Latitude	Hemoparasites				
				Prevalence (%)	N (Inf.)	Cluster 1	Cluster 2	N (Non-Inf.)
Isabela	*A. c. cristatus*	−91.4246	−0.78524	3.45	1	1	0	28
Floreana	*A. c. venustissimus*	−90.50911	−1.31968	6.67	2	2	0	28
Punta Pitt (SC)	*A. c. godzilla*	−89.24174	−0.71432	13.33	4	4	0	26
**Santa Fé**	***A. c. trillmichi***	**−90.02861**	**−0.82581**	**16.67**	**5**	**3**	**2**	**25**
**Fernandina**	***A. c. cristatus***	**−91.38947**	**−0.44264**	**20**	**6**	**5**	**1**	**24**
Santa Cruz	*A. c. hassi*	−90.30732	−0.7418	20.59	7	7	0	27
**Genovesa**	***A. c. nanus***	**−89.97349**	**0.31065**	**26.67**	**8**	**6**	**2**	**22**
**Pinta**	***A. c. sielmanni***	**−90.73948**	**0.5434**	**26.67**	**8**	**6**	**2**	**22**
Santiago	*A. c. wikelskii*	−90.86495	−0.24215	33.33	10	10	0	20
Isla Lobos (SC)	*A. c. mertensi*	−89.5688	−0.85643	33.33	10	10	0	20
Española	*A. c. venustissimus*	−89.62029	−1.39502	36.67	11	11	0	19
**Marchena**	***A. c. hayampi***	**−90.50774**	**0.30051**	**40.74**	**11**	**6**	**5**	**16**
La Lobería (SC)	*A. c. mertensi*	−89.62125	−0.92214	50	15	15	0	15

**Table 2:** Results from the logistic regression to explore the relationship between the probability of individual infection and the different populations of origin (Isabela is set as a reference level).

	Estimate	Std. Error	z-Value	Pr(>|z|)
(Intercept)	−3.332	1.017	−3.276	**0.001**
Santa Cruz	1.982	1.102	1.799	0.072
Española	2.786	1.085	2.567	**0.010**
Fernandina	1.946	1.115	1.746	0.081
Floreana	0.693	1.253	0.553	0.580
Genovesa	2.321	1.098	2.114	**0.035**
Marchena	2.958	1.090	2.714	**0.007**
Pinta	2.321	1.098	2.114	**0.035**
Santiago	2.639	1.088	2.425	**0.015**
Santa Fe	1.723	1.129	1.526	0.127
Isla Lobos (San Cristóbal)	2.639	1.088	2.425	**0.015**
La Lobería (San Cristóbal)	3.332	1.081	3.084	**0.002**
Punta Pitt (San Cristóbal)	1.460	1.150	1.270	0.204

*p*-values (*p* < 0.05) are highlighted in bold.

**Table 3:** Sex comparison in the number of infected and non-infected marine iguanas by population. Fisher’s exact test (*p*-value) for each island is shown (significant values are highlighted in bold. Only males and females are considered (*n* = 356).

	Males		Females		Fisher’s Exact Test
	Inf	Non-Inf	Inf	Non-Inf	*p*-Value
Santa Cruz	2	14	4	10	0.3778
Española	5	11	5	8	0.7141
Fernandina	0	15	6	9	**0.01686**
Floreana	0	15	2	12	0.2241
Genovesa	2	7	6	15	1
Isabela	0	20	1	5	0.2308
Marchena	7	11	2	3	1
Pinta	1	6	7	15	0.6349
Santiago	4	13	6	7	0.2553
Santa Fe	3	13	2	10	1
Isla Lobos-San Cristóbal	4	4	1	5	0.3007
Lobería-San Cristóbal	8	12	7	3	0.2451
Punta Pitt-San Cristóbal	1	19	2	6	0.188

**Table 4:** Number of infected and uninfected specimens for different combinations of ticks and their hosts (paired data). Numbers correspond to combinations of tick-host for the same individual (i.e., “Marine iguana Infected” and “Tick Infected” represent marine iguana individuals infected by hemoparasites in both the blood samples and the ticks collected from the same specimens).

	Marine Iguana	
	Inf	Non-Inf
Tick		
Inf	8	14
Non-Inf	10	43

## Data Availability

All the sequences generated in this study have been submitted to GenBank (accession numbers: ON220615-ON220734). Other relevant data can be found in the manuscript, the Supplementary Material or can be obtained from the authors.

## Supplementary Materials

The following supporting information can be downloaded at: https://www.mdpi.com/article/10.3390/ani12091142/s1. Supplementary File S1: Result from the BLAST analysis. Supplementary File S2: Metadata and accession numbers of samples from this study.
